# Microvascular and structural analysis of the retina and choroid in heart failure patients with reduced ejection fraction

**DOI:** 10.1038/s41598-023-32751-w

**Published:** 2023-04-04

**Authors:** Ehsan Khalilipur, Zahra Mahdizad, Negin Molazadeh, Hooshang Faghihi, Nasim Naderi, Mohammadreza Mehrabi Bahar, Ata Firouzi, Parham Sadeghipour, Majid Maleki, Sahel Soltani Shahgoli, Elias Khalili Pour, Hamid Riazi-Esfahani

**Affiliations:** 1 Cardiovascular Intervention Research Center, Rajaie Cardiovascular, Medical and Research Center, Iran University of Medical Sciences, Tehran, Iran; 2 Retina Ward, Farabi Eye Hospital, Tehran University of Medical Sciences, Qazvin Square, South Karegar Street, Tehran, 1336616351 Iran

**Keywords:** Cardiology, Biomarkers, Medical research, Eye diseases

## Abstract

This cross-sectional study was designed to assess alterations of choroidal and retinal microvasculature in patients with Heart Failure with Reduced Ejection Fraction (HFrEF) and compare them with a normal age and sex-matched population. Fifty-two eyes of 26 patients with HFrEF (left ventricular ejection fraction [LVEF] < 40%) and 64 eyes of 32 healthy individuals were considered as the patient and the control groups, respectively. We found no statistically significant differences in age-adjusted mean central macular thickness (CMT), superficial or deep retinal capillary plexus vascular densities, and choriocapillaris flow (CC flow) density between the HFrEF group and the normal controls, with the exception of the parafoveal mean superficial capillary plexus vascular density (P = 0.023), which remained statistically significant after adjusting for age (P = 0.034). The patients with HFrEF had a significantly lower subfoveal choroidal thickness (SFCT) than the normal subjects (264 ± 82 vs 313 ± 72; *P* = 0.009), and the difference was still statistically significant after age adjustment (*P* = 0.026). Although choroidal vascularity index (CVI) was lower in the HFrEF group than in the control group, the difference was not statistically significant before and after age adjustment (73.45 ± 6.67 vs 75.77 ± 5.92; *P* = 0.118 and *P* = 0.096, respectively). In conclusion, in patients with HFrEF, we observed a reduction in parafoveal retinal VD in the superficial capillary plexus, as well as SFCT, but no significant change in CVI, CMT, or CC flow density.

## Introduction

Heart failure with reduced ejection fraction (HFrEF) is a complicated clinical condition associated with high morbidity and mortality, with a 1-year death rate of 7.2% and a 1-year hospitalization rate of 31.9% in chronic HF patients. In hospitalized patients with acute HF, these rates rise to 17.4% and 43.9%, respectively^1^.

HFrEF is also accompanied by a systemic response attempting to compensate for the insufficiency^2^. Multiple investigations have demonstrated a possible association between HFrEF and other peripheral vessels in the human body, including the cerebral, renal, and ocular vasculatures^3^.

The retina has the highest oxygen consumption per volume in the body, and it is supplied with blood from 2 sources: the inner retina receives capillaries from the central retinal artery through the superficial and deep capillary plexuses, while the outer retina, which is mainly non-vascular, is supplied by the choroid^4,5^.

The choroid, a richly vascularized structure with the highest blood flow, provides most of the intraocular blood flow and supplies the energy demands of the outer retina and the retinal pigment epithelium^6^.

The development of enhanced depth imaging optical coherence tomography (EDI-OCT) and optical coherence tomography angiography (OCTA) has enabled sufficient, noninvasive, and repeatable in-depth analyses of retinal and choroidal structures as well as blood flow in comparison with previous imaging techniques such as ultrasonography and fluorescein/indocyanine green angiography^7,8^.

Accumulating evidence supports the notion that the retinal and choroidal microvasculature can be indicators or potential biomarkers of systemic cardiovascular diseases and the future direction of their severity, as well as a therapeutic index and an emerging endpoint of target organ damage^9^.

In this cross-sectional study, we aimed to evaluate alterations in central macular and choroidal thickness (CT), the choroidal vascularity index (CVI), and chorioretinal microvascular density using OCTA in patients with HFrEF and then compare them with a normal age-matched population.

## Methods

### Subjects

This cross-sectional study recruited patients with a diagnosis of HFrEF (left ventricular ejection fraction [LVEF] < 40%) who were referred from the Rajaie Cardiovascular Medical and Research Center to the Farabi Eye Hospital between November 2021 and March 2022. All patients were managed with guideline-directed medical therapy, and all patients had been visited regularly to uptitrate the guideline directed medical therapy to the dose they could tolerate and none was in acute decompensated HF^10^.

This study was approved by the institutional review board of Iran University of Medical Sciences, Tehran, Iran. The study protocol adhered to the tenets of the Declaration of Helsinki, and all participants gave written informed consent before entering into the study.

An age and sex-matched control group was selected from individuals who underwent routine ocular examinations at our clinic. They all had unremarkable ocular examinations and no history of ocular or cardiac diseases.

Exclusion criteria were the presence of acute HF or any acute cardiac condition (e.g., recent myocardial infarction, recent hospitalization within 4 weeks, any recent dysrhythmia with hospitalization, and a history of pulmonary embolism), a history of retinal vascular diseases including diabetic retinopathy, retinal artery or vein occlusion or insufficiency, myopic retinopathy, retinal dystrophy, glaucoma, uveitis, age-related macular degeneration, any retinal surgery or laser photocoagulation, systemic or ocular diseases that prevented ocular examinations, and severe media opacity, which might affect the image quality. Additionally, patients who had recently used a vasoconstrictive medication or had any associated systemic disorders that may affect the eye (e.g., diabetes, systemic corticosteroid use, and poorly controlled hypertension which defined as systolic blood pressure ≥ 140 mmHg and/or a diastolic blood pressure ≥ 90 mmHg) were also excluded from both the patient and control groups. Patients were included if they had a refractive error between − 6 and + 6 diopter and a best-corrected visual acuity of 20/25 or better.

Clinical parameters analyzed in the patients included LVEF measured in transthoracic echocardiography or cardiac MRI and systolic and diastolic blood pressures. In the HFrEF group, cardiomyopathy was categorized as ischemic and dilated.

The patients underwent thorough ophthalmic examinations, including slit-lamp biomicroscopy and dilated indirect ophthalmoscopy. A masked optometrist measured the best-corrected visual acuity using a Snellen chart, and the results were then converted into the logarithm of the minimum angle of resolution.

### Image acquisition and processing

En face OCT-A and EDI-OCT images were obtained from macula using the RTVue XR 100 Avanti instrument (Optovue, Inc, Fremont, CA, USA) for all the patients.

For OCT-A acquisition, a 6 × 6 mm fovea-centered image was obtained from each eye. A quality score of more than 5/10 (according to the built-in RTVue software quality assessment report) was set as the minimum quality requirement, and imaging was repeated until this goal was achieved. Eyes with low image quality or different artifacts, including defocusing, movement, shadow, and decentration, preventing the accurate measurement of vascular density (VD) were excluded. With the aid of the integral module in the Angio Analytics software (version 2017.1.0.151), the different retinal layers were segmented automatically. All automated segmentations of retinal layers in OCTA images were rechecked and segmentation errors were manually corrected by two retina experts (EKP and HRE). The central macular thickness (CMT), densities of the superficial and deep capillary plexuses, and also choriocapillaris flow density (CC flow) were calculated using the built-in software. Retinal and choriocapillaris VD was calculated as a percentage of the area occupied by flowing blood vessels in the selected area of each layer.

**Figure 1:**
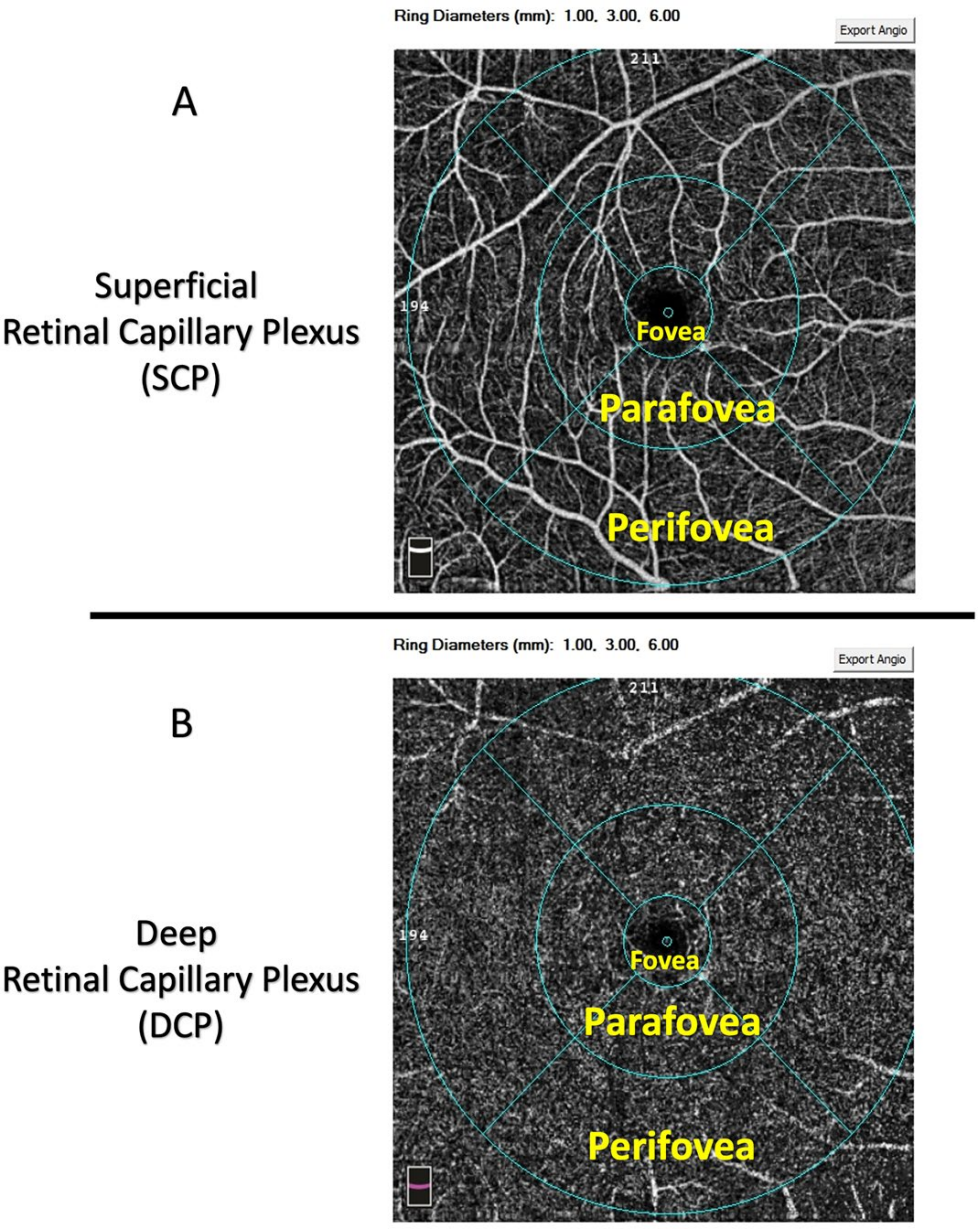
The images depict the density maps of (**A**) the superficial capillary plexus (SCP) and (**B**) the deep capillary plexus (DCP). The blue circles over the images are 1-, 3-, and 6-mm circles that represent the foveal, parafoveal, and perifoveal regions, respectively. The tables beside each region show the density measurement in the different sectors of SCP and DCP.

The foveal region was defined as a circle with a 1 mm diameter surrounded by the parafoveal region, defined as a ring with an internal diameter of 1 mm and an external diameter of 3 mm, and the perifoveal region, defined as a ring with an internal diameter of 3 mm and an external diameter of 6 mm (Fig. 1A,B). Measurements are taken in the superficial capillary plexus comprised whole superficial VD, foveal superficial VD, parafoveal superficial VD, and perifoveal superficial VD. Measurements obtained in the deep capillary plexus consisted of whole deep VD, foveal deep VD, parafoveal deep VD, and perifoveal deep VD.

For the measurement of CMT, SFCT, and CVI on EDI-OCT acquisition, the patients were positioned appropriately, and fovea-centered OCT B-scans at 8 mm × 12 mm raster patterns were captured. As the choroidal structures exhibit diurnal variations, all EDI-OCT scans were performed between 8:00 AM and 11:30 AM^11^.

Only complete, well-centered scans with at least a 6-signal strength value and no motion or blinking artifacts were accepted. After each assessment, 2 masked, independent reviewers assessed the best image on a computer screen. When the 2 graders determined that both the inner and outer borders of the choroid were distinguishable, the image was captured and used for analysis.

**Figure 2:**
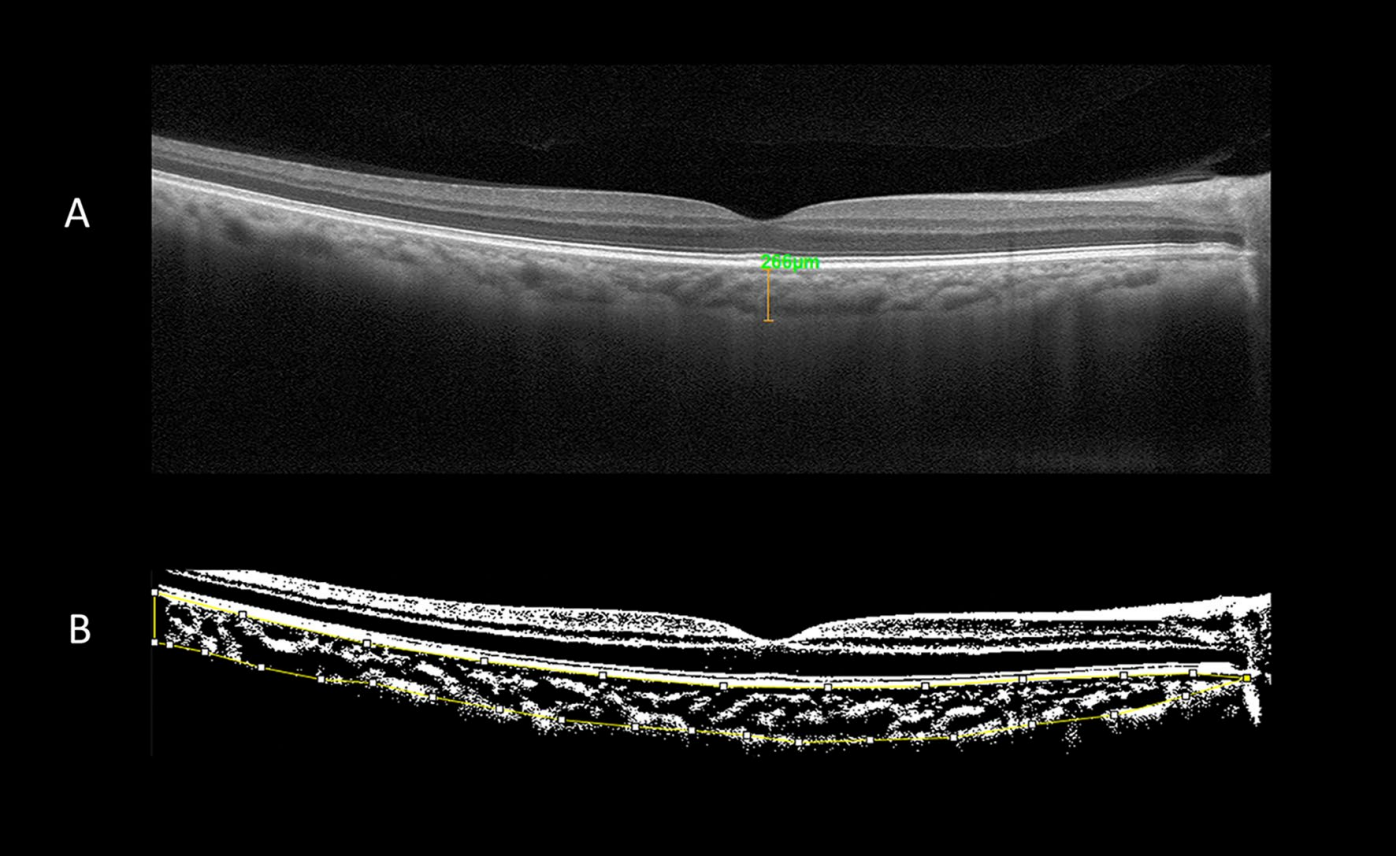
(**A**) A fovea-centered enhanced depth imaging optical coherence tomography image of a patient was used for subfoveal choroidal thickness measurement. With the aid of the caliper function of the AngioVue software, the subfoveal choroidal thickness was measured as the vertical distance between the hyper-reflective line of the Bruch membrane and the inner surface of the sclera. (**B**) The choroidal vascularity index was measured using the FIJI software. The light pixels in the selected choroidal region were classified as choroidal stroma or interstitial area, whereas the dark pixels were classified as the luminance area. The ratio of the luminance area to the total choroidal area is referred to here as the choroidal vascularity index.

With the use of the caliper function of the AngioVue software the SFCT was measured as previously stated in other research: the vertical distance between the hyper-reflective line of the Bruch membrane and the inner surface of the sclera (Fig. 2A). Multiple studies have demonstrated high degrees of intersystem, interobserver, and intervisit repeatability for SFCT readings beneath the fovea. Every value was measured twice, and the mean was then determined^12,13^.

CVI was measured using the method described by Agrawal et al.^14^ in FIJI (an extended version of the ImageJ software, version 1.51 h; National Institutes of Health, Bethesda, Maryland; accessible at http://imagej.nih.gov/Fiji/). Further, the total choroidal area, the luminance area, and the stromal area were determined (Supplement 1). The ratio of the luminance area to the total choroidal area is referred to here as CVI (Fig. 2B).

All manual segmentations, including the delineation of the sclerochoroidal junction and the measurement of CT, were conducted by a skilled grader (ZM) and rechecked by another independent grader (EKH). In the case of any dispute, the outlines were segmented by consensus.

The inter-rater reliability of the CMT, SFCT, and CVI measurements was evaluated using the absolute agreement model of the interclass correlation coefficient on 20 EDI-OCT images, initially segmented by the 2 independent graders^15^.

### Statistical analysis

Quantitative data are presented as the mean ± the standard deviation and qualitative data as counts and percentages. The parameters were compared between the 2 groups using the *t* test and the χ^2^ test, whenever appropriate. In addition, the generalized estimating equation was employed to consider the probable correlation of the measurements between the 2 eyes of a subject^16–18^. Another generalized estimating equation analysis was applied to adjust for the possible confounders. All the statistical analyses were performed using the SPSS software (IBM Corp, released 2019; IBM SPSS Statistics for Windows, version 25.0. Armonk, NY: IBM Corp). A *P* value of less than 0.05 was considered statistically significant.

## Results

The current study included 52 eyes of 26 patients with HFrEF as the patient group and 64 eyes of 32 healthy individuals as the control group. The demographic and clinical characteristics of both groups are presented in Table 1. There were no statistical differences between the 2 groups in terms of their mean age, sex, best-corrected visual acuity, spherical refraction, and systolic and diastolic pressure at the time of examination. The mean LVEF in the HFrEF group and the control group was 25 ± 13 and 53 ± 11%, respectively (*P* = 0.021). None of the patients in the control group had symptoms relevant to a diagnosis of HF with preserved EF.

**Table 1:** The demographic and clinical characteristics of both groups.

Variables	HFREF group	Control group	*P* value*
Age (y)	53 ± 13 (31 to 83)	51 ± 15 (16–79)	0.639
Sex (female/male)	0.4 12/40	0.7 24/40	0.268
BCVA (LogMAR)	0.11 ± 0.19 (0 to 0.92)	0.04 ± 0.09 (0 – 0.36)	0.111
Spherical refraction (D)	0.02 ± 1.27 (− 5 to 2.5)	0.02 ± 0.83 (− 2.5 to 2)	0.996
HFrEF category			
DCM	14 (53.8%)		
ICMP	12 (46.2%)		
LVEF (%)	25 ± 13 (10–39)	53 ± 11 (50–65)	0.021
Systolic BP (mm Hg)	112 ± 13 (90–130)	123 ± 8 (90–138)	0.351
Diastolic BP (mm Hg)	76 ± 12 (60–100)	72 ± 13 (60–95)	0.263

*DCM* dilated cardiomyopathy, *HFrEF* heart failure with reduced ejection fraction, *ICMP* ischemic cardiomyopathy, *LogMAR* logarithm of the minimum angle of resolution, *BP* blood pressure.

*P values were calculated using the *t* test.

The whole image age-adjusted mean retinal superficial and deep vascular plexus densities in the HFrEF patients was lower than that of the normal control group although was not statistically significant (47.6 ± 3.6 vs 49.7 ± 3.6; *P* = 0.162 and 47.9 ± 6 vs 49.4 ± 6.3; *P* = 0.374, respectively). Also, foveal and perifoveal age-adjusted mean superficial and deep capillary plexus densities did not show statistically significant differences between the HFrEF group and the normal controls (*P* = 0.650, *P* = 0.220, *P* = 0.147, *P* = 0.361, respectively).

**Figure 3:**
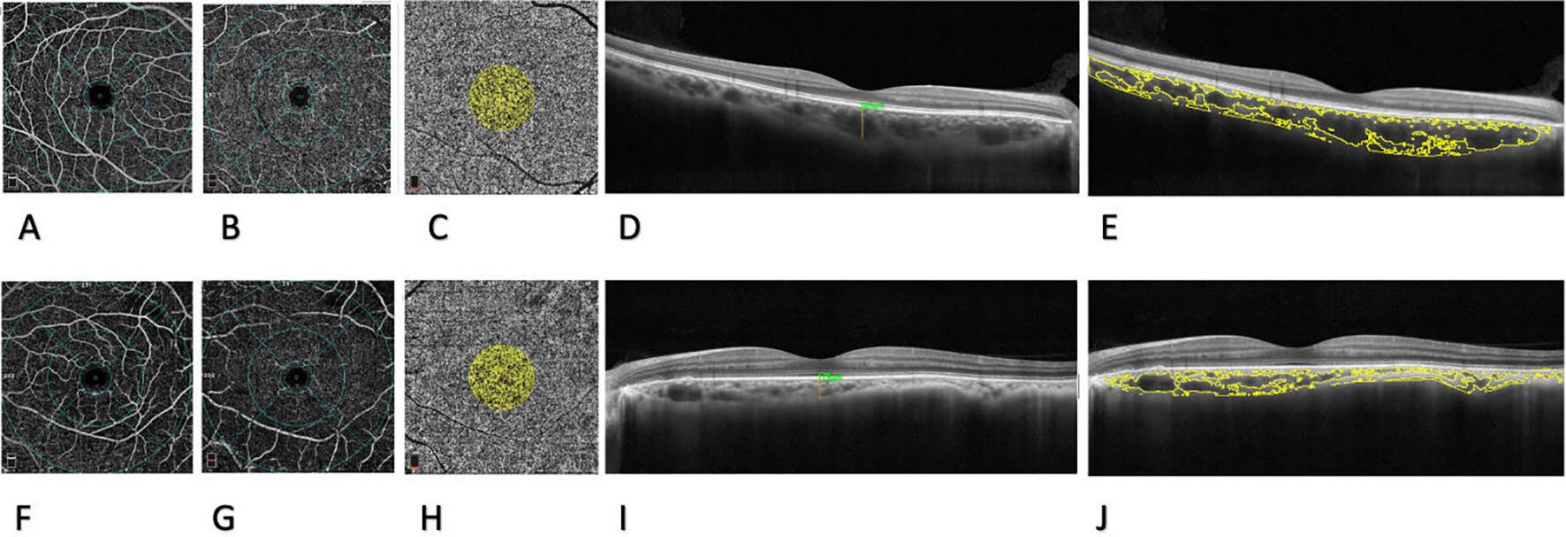
Optical coherence tomography angiography (OCTA) maps of the superficial (**A**), deep retinal capillary plexus (**B**), and choriocapillaris flow (**C**) in a 30-year-old healthy participant included in the current investigation. Corresponding cross-sectional macular optical coherence tomography (OCT) was utilized for the measurement of subfoveal choroidal thickness (SFCT) (**D**) and choroidal vascular index (CVI) (**E**). OCTA maps of the superficial (**F**), deep retinal capillary plexus (**G**), and choriocapillaris flow (**H**) in a 41-year-old patient with Heart Failure with Reduced Ejection Fraction (HFrEF) included in the current study. The patient's corresponding cross-sectional macular optical coherence tomography (OCT) is used to calculate subfoveal choroidal thickness (SFCT) (**H**) and choroidal vascular index (CVI) (**J**).

Parafoveal mean superficial capillary plexus densities showed statistically significant differences between the HFrEF group and the normal controls (47.7 ± 7.6 vs 51.1 ± 5.1; *P* = 0.023), and the difference remained statistically significant after adjusting for age (*P* = 0.034). But, parafoveal age-adjusted mean deep capillary plexus densities did not show statistically significant differences between the HFrEF group and the normal controls (52.4 ± 4.6 vs 53.4 ± 5.7; *P* = 0.499). The mean superficial and deep retinal vessel densities of both groups are shown in Table 2 and Fig. 3.

**Table 2:** The mean superficial and deep retinal vessel densities of both groups.

	HFrEF group	Control group	Diff	95% confidence interval		*P* value*	Age- and sex-adjusted *P* value*
				Lower	Upper		
Superficial vessel density							
Whole image	47.6 ± 3.6	49.7 ± 3.6	− 1.267	− 3.044	0.509	0.104	0.162
Fovea	20.5 ± 7.8	21 ± 8.5	− 0.767	− 4.075	2.541	0.751	0.650
Parafovea	47.7 ± 7.6	51.1 ± 5.1	− 3.049	− 5.868	− 0.230	**0.023**	**0.034**
Perifovea	48.4 ± 3.9	49.8 ± 3.9	− 1.179	− 3.066	0.707	0.153	0.220
Deep vessel density							
Whole image	47.9 ± 6	49.4 ± 6.3	− 1.243	− 3.986	1.500	0.270	0.374
Fovea	36.6 ± 8	38.9 ± 8.6	− 2.585	− 6.076	0.907	0.213	0.147
Parafovea	52.4 ± 4.6	53.4 ± 5.7	− 0.778	− 3.035	1.479	0.350	0.499
Perifovea	49 ± 6.8	50.8 ± 6.9	− 1.434	− 4.514	1.645	0.248	0.361

Significant values are in [bold].

*HFrEF* heart failure with reduced ejection fraction.

*P values were calculated using the generalized estimating equation.

The inter-class correlation coefficients for SFCT and CVI measurements were 0.98 (95% CI 0.97–0.99) and 0.96 (95% CI 0.91–0.98), respectively. The mean central macular thickness (CMT), Choriocapillaris flow density (CC flow), SFCT, and CVI of both groups are shown in Table 3. Age and sex-adjusted central macular thickness (CMT) was not statistically significant between the patients with HFrEF and normal subjects (256 ± 23 vs 253 ± 16; *P* = 0.592). Also, age and sex-adjusted Choriocapillaris flow (CC flow) was not statistically significant between the patients with HFrEF and normal subjects (0.65 ± 0.05 vs 0.65 ± 0.04; P = 0.660). The patients with HFrEF had a significantly lower SFCT than the normal subjects (264 ± 82 vs 313 ± 72; *P* = 0.009), and the difference was still statistically significant after age adjustment (*P* = 0.026). Although CVI was lower in the HFrEF group than in the control group, the difference was not statistically significant before and after age adjustment (73.45 ± 6.67 vs 75.77 ± 5.92; *P* = 0.118 and *P* = 0.096, respectively) (Fig. 3).

**Table 3:** The mean CC flow, SFCT, and CVI of both groups.

	HFrEF group	Control group	Diff	95% confidence interval		*P* value*	Age- and sex-adjusted *P* value*
				Lower	Upper		
CMT	256 ± 23	253 ± 16	2.515	− 6.122	13.471	0.462	0.592
CC flow	0.65 ± 0.05	0.65 ± 0.04	0.006	− 0.021	0.033	0.965	0.660
SFCT	264 ± 82	313 ± 72	− 38.842	− 73.073	− 4.610	**0.009**	**0.026**
CVI	73.45 ± 6.67	75.77 ± 5.92	− 2.504	− 5.450	0.442	0.118	0.096

Significant values are in [bold].

*HFrEF* heart failure with reduced ejection fraction, *CMT* central macular thickness, *CC* flow: choriocapillaris flow, *SFCT* subfoveal choroidal thickness, *CVI* choroidal vascularity index.

*P values were calculated using the generalized estimating equation.

In the patient group, SFCT was not correlated with LVEF, systolic blood pressure, and diastolic blood pressure (*P* = 0.845, *P* = 0.930, and *P* = 0.123, respectively). In the patient group also, CVI was not associated with LVEF, systolic blood pressure, and diastolic blood pressure (*P* = 0.430, *P* = 0.413, and *P* = 0.776, respectively). Also in the normal group, we could not find any correlation between SFCT or CVI and cardiovascular factors like LVEF, systolic blood pressure, and diastolic blood pressure. The analyses also showed no statistical differences in relation to the type of cardiomyopathy in the HFrEF group (*P* = 0.063). Subgroup analysis based on the type of cardiomyopathy (ischemic or dilated) revealed no statistically significant differences between the two groups in terms of microvascular and structural characteristics of the retina and choroid (P > 0.05 for all).

## Discussion

Although numerous clinical markers and risk factors have been identified, novel clinical markers and approaches are required to assess patient risk stratification with HFrEF. Relying solely on retinal vasculature as a biomarker can complicate the evaluation of patients with HF. On the other hand, the ability to examine the choroidal layers with swept source or EDI-OCT offers new research opportunities for patients with HFrEF and the ischemic type of HFrEF, which is the leading cause of LV dysfunction in adults worldwide^19–22^.

SFCT is commonly used in research trials. Still, it reflects only the whole choroidal structure and may be affected by various factors such as age, axial length, intraocular pressure, and systolic blood pressure^23–25^. It also fails to distinguish vascular from stromal components^26^. Therefore, since 2014, the focus of investigations has been switched to finding a method for choroidal structure analysis with a better distinction between the lumen and the stroma^27^. The term “CVI” was introduced as the ratio of the luminance area to the total choroidal area and was reported to be around 65% in healthy eyes^14^. It has been proved that CVI is a more informative, reliable, and steady index of proportional change in the choroidal vasculature as it is relatively resistant to changes in other physiological parameters^14,28^.

Similar to other organs, the choroid could be susceptible to any chronic cardiac processes. Extreme hypertensive retinopathy is associated with hypertensive choroidopathy and choroidal thickening. Hypercholesterolemia is linked to choroidal thickening, whereas smoking, ocular ischemia syndrome, and systemic hypertension are linked to a thinner choroid. Alternatively, data addressing the association between CT and carotid artery stenosis and diabetes mellitus are conflicting^9,29–33^.

Comparing 56 eyes of 56 patients with chronic HF with 56 eyes of 56 individuals of the same sex, Altinkaynak et al.^34^ discovered that the mean SFCT value was statistically considerably lower in patients with chronic HF, and it was positively correlated with EF. They hypothesized that a low cardiac output might lead to vasoconstriction development in peripheral vessels like orbital and choroidal arteries to maintain critical organs’ blood supply, causing choroidal ischemia and retinal pigment epithelium atrophy. Rakusiewicz et al.^21^ discovered that children with congestive HF due to dilated cardiomyopathy had a thinner CT at all measured locations. They found that chronic HF impacted CT and that this parameter might be useful for monitoring the clinical progression of dilated cardiomyopathy in children. Nonetheless, another study by Alur et al.^35^ found neither a significant difference in SFCT between patients and controls nor a correlation between SFCT and EF. Although there is no clear explanation for the divergent results, they may be explained by the wide range of EF, dissimilar treatments received by each study’s patients, and the impact of diuretics and angiotensin-converting enzyme inhibitors on SFCT^29^.

HFrEF is accompanied by peripheral vasoconstriction, which ensures appropriate perfusion and oxygenation to the heart, brain, and other vital tissues^3,36^. It is suggested that this compensatory vasoconstriction affects the choroidal arteries and, thus, lowers SFCT^34^. When choroidal vasoconstriction persists, the ensuing chronic ischemia may result in the atrophy of the retinal pigmented epithelium, which exacerbates SFCT reduction^2,29,32,33^. Consequently, as shown in the current study, HFrEF can be represented in choroidal thinning, and HF can be reflected in the thinning of the choroid.

Regardless of SFCT abnormalities in individuals with HFrEF, it is unclear which component of the choroid (the stroma or the lumen of the choroidal vessels) is most affected by HFrEF. To our knowledge, the present study is the first to evaluate CVI in the setting of HFrEF. We demonstrated that CVI was decreased in the HFrEF group, but the association was not statistically significant before and after age adjustment (*P* = 0.118 and *P* = 0.098, respectively), suggesting that diminishing CT in HFrEF can be associated with the shrinkage of both components of the choroid (i.e., the stroma and the lumen).

Any defect or disruption in the choroid or its blood flow may result in degenerative changes and neovascularization^25^. In contrast to the retinal vasculature, which utilizes autoregulation to maintain relatively constant blood flow despite variations in ocular perfusion pressure, the regulation of blood flow in the choroid is complex^37^. It is now well established that the choroid is not a passive vascular bed in which a decrease in perfusion pressure leads to a linear decrease in blood flow^6,37,38^. The choroid has neural control and is supplied by sympathetic and parasympathetic nerve fibers, which may help the choroid to compensate for blood pressure alterations through vasoconstriction or dilation^39,40^. There is also a compensatory myogenic mechanism in the choroid: smaller resistance arteries can change smooth muscle contraction and tone to maintain vessel wall stretch in the desired range^40^. It seems that the choroid has a weak but significant ability to autoregulate its blood flow in response to changes in ocular perfusion pressure^41^.

Previous studies have reported that patients with HF have reduced retinal vessel density compared with the normal population^42^. It has been hypothesized that the mentioned peripheral vasoconstriction in response to a low cardiac output is the pathophysiological mechanism that triggers this finding^42–44^. Therefore, retinal vessel density as measured by OCTA may provide an insight into the global microperfusion and hemodynamic state of patients with chronic HF^22,45^. In the current study, except for the parafoveal mean retinal superficial capillary plexus vascular density, we found no statistically significant differences in age-adjusted mean central macular thickness (CMT), superficial or deep retinal capillary plexus vascular densities and choriocapillaris flow density (CC flow) between the HFrEF group and the normal controls. Prior to the current investigation, very few OCTA evaluations of choroidal or retinal superficial and deep capillary plexus densities in patients with heart failure have been conducted. Rakusiewicz et al. found that children with congestive HF due to dilated cardiomyopathy had reduced superficial and deep capillary plexus densities at all assessed locations. They discovered that chronic HF affected retinal vascular density and those retinal vascular parameters may be relevant for monitoring the clinical course of pediatric dilated cardiomyopathy^22^. Although there is no clear explanation for the discrepancies between our results and those of other studies, they may be due to the wide range of EF and age of patients, the different treatments received by the patients in each study, and the effect of diuretics and angiotensin-converting enzyme inhibitors on retinal vascular density^22,46^. Since the blood supply to the retina is physiologically kept constant by specific autoregulation mechanisms, such a decrease in parafoveal superficial VD in patients with chronic HF can also be justified by insufficiency to autoregulate the retinal blood supply in these patients^22^.

The main advantage of our study is its use of a noninvasive technology that enables the reproducible and quantitative evaluation of abnormalities in the retinal and choroidal microvasculature in a homogeneous group of patients with HFrEF. This study does come, however, with several limitations. Firstly, the relatively small sample size might affect the results. Secondly, the cross-sectional design of the current study precluded longitudinal change assessment in the retinal and choroidal vasculatures. Thirdly, the study was conducted in a tertiary center, which may have led to the recruitment of more chronic or complicated patients and, thus, selection bias. Fourthly, the duration of chronic HF was not specified, which might have affected the results. Finally, the influence of individually started HF treatment on the obtained results concerning retinal VD and the choroidal vasculature should not be ruled out.

We believe that our findings demonstrate a new multidisciplinary pathway in the diagnosis and management of HFrEF, which is a syndrome affecting literally every organ in the human body. Accordingly, we advocate retinal and choroidal vasculature examination as a promising endpoint in future studies.

In conclusion, we demonstrated a reduction in parafoveal superficial retinal VD as well as SFCT in patients with HFrEF, although CMT and CVI were not changed significantly. Further long-term observations of larger numbers of patients will determine whether these biomarkers may be useful in clinical practice in patients with HFrEF.

## Supplementary Information

Supplementary Information.

## Data Availability

The datasets used and/or analyzed during the current study available from the corresponding author on reasonable request.
