# Supplementary material for: Microvascular and structural analysis of the retina and choroid in heart failure patients with reduced ejection fraction
Source: Sci Rep. 2023 Apr 4;13:5467. doi: 10.1038/s41598-023-32751-w (PMC10073248; doi:10.1038/s41598-023-32751-w)
Supplement: Supplementary file 1 — Supplementary Information. [file 41598_2023_32751_MOESM1_ESM.docx]

**Supplement -1**

FIJI (an extended version of ImageJ software, version 1.51h; National Institutes of Health, Bethesda, Maryland; accessible at http://imagej.nih.gov/Fiji/) was used for image analysis. The choroid's borders were then selected using the software's free hand tool. The nasal margin was the temporal edge of the optic nerve head, and the temporal margin was 8 mm from the temporal edge of the optic nerve head. As previously disclosed, a modified Niblack approach was utilized to binarize choroidal area in OCT images.^1^ Briefly, three choroidal vessels with lumens larger than 100 microns were chosen at random using the oval selection tool of the toolbar, and the average reflectance of these areas was calculated using software. To reduce noise in the OCT image, the average brightness was chosen as the minimal value. In the OCT image, the ROI was selected and set by the ROI manager. The image was then converted to 8 bits and modified using Niblack's auto local threshold. The binarized image was reconverted to an RGB image, and the luminance region was identified using the color thresholding tool. The light pixels were classified as choroidal stroma or interstitial area, whereas the dark pixels were classified as the luminance area (LA). Total Choroidal Area (TCA), luminance area (LA), and stromal area (SA) were determined automatically. The ratio of LA to TCA is referred to here as the choroidal vascular index (CVI) (Figure-2-B).

1. Agrawal, R. *et al.* Choroidal vascularity index as a measure of vascular status of the choroid: Measurements in healthy eyes from a population-based study. *Sci. Rep.* **6**, 21090 (2016).
